# Editorial: Vulvodynia and beyond: innate immune sensing, microbes, inflammation, and chronic pain

**DOI:** 10.3389/fcimb.2023.1338659

**Published:** 2023-12-08

**Authors:** Megan L. Falsetta

**Affiliations:** ^1^ Department of Obstetrics and Gynecology, University of Rochester, Rochester, NY, United States; ^2^ Department of Pharmacology and Physiology, University of Rochester, Rochester, NY, United States

**Keywords:** pain, vulva, vestibulitis, vulvodynia, inflammation, TRPV cation channels, lipidome

Although it is inevitable that every person with a vulva will experience pain or discomfort in this area at some point in their life, vulvar disease remains inadequately understood (Damsted-Petersen et al., 2009; Bohm-Starke, 2010; Reed et al., 2014; Akopians and Rapkin, 2015; Bornstein et al., 2016; Falsetta et al., 2017; Stenson, 2017; Bergeron et al., 2020; Graziottin et al., 2020; Vasileva et al., 2020; Bekauri et al., 2023; Falsetta et al., 2023). Patients with vulvodynia, or chronic vulvar pain, suffer from burning, stabbing, or “knife-like” pain that is poorly explained and recalcitrant to treatment (Haefner et al., 2005; Stockdale and Lawson, 2014). This pain occurs in the absence of any obvious disease pathology, making it a diagnosis of exclusion. Thus, patients will suffer, at minimum, months and most often, years before they are diagnosed. Treatment requires an escalation of interventions, starting with changes to hygiene practices, progressing to topical lidocaine and oral therapies such as gabapentin, eventually resulting in surgical removal of the vestibule (ring of tissue surrounding the vaginal opening) (Fischer, 2004; Danby and Margesson, 2010; Nunns et al., 2010; Tieu and MacGregor, 2011; Stockdale and Lawson, 2014; Eppsteiner et al., 2014; Sadownik, 2014; Goldstein et al., 2016; Falsetta et al., 2017; Brown et al., 2018; Vasileva et al., 2020; Bohm-Starke et al., 2022; Falsetta et al., 2023). However, some patients experience generalized pain affecting the whole vulva, such that surgical intervention is not possible.

Patients with vulvar pain have a low quality of life, and disease is often accompanied by depression, pelvic floor dysfunction, relationship issues, and other comorbidities (Arnold et al., 2006; Reed et al., 2012; Reed et al., 2014; Sadownik, 2014; Vasileva et al., 2020; Rubal et al., 2023). While it is socially acceptable to discuss pain in other parts of the body, there is significant stigma associated with vulvar pain or “woman troubles” (Nguyen et al., 2013). Patients often feel isolated, invalidated, or even that they are being “gaslighted” by the medical community and that their experiences are not being taken seriously (Bergeron et al., 2014; Niedenfuehr et al., 2023). It has been recognized that screening, diagnosis, and intervention must be improved for vulvar disease, yet women’s health research as a whole has been chronically underfunded (Reporter, 2023; Smith, 2023).

Advances in the field, roughly within the last decade, have led to the creation of rodent and cell culture models that have facilitated greater mechanistic understanding of disease, while enabling screening of new therapies and therapeutic targets (Gordon et al., 2003; Gerber et al., 2008; Farmer et al., 2011; Leclair et al., 2011; Leclair et al., 2013; Martinov et al., 2013; Leclair et al., 2014; Akopians and Rapkin, 2015; Ali et al., 2015; Falsetta et al., 2015; Foster et al., 2015; Tommola et al., 2015; Akbar et al., 2016; Chalmers et al., 2016; Falsetta et al., 2016; Tommola et al., 2016; Falsetta et al., 2017; Harlow et al., 2017; Falsetta et al., 2018; Zanotta et al., 2018; Arriaga-Gomez et al., 2019; Barry et al., 2019; Bedford et al., 2020; Bergeron et al., 2020; Falsetta et al., 2021; Rangappa et al., 2021; Awad-Igbaria et al., 2022; Falsetta et al., 2023). However, it is still not clear if vulvodynia is a singular disease with different manifestations or multiple related diseases that are grouped simply because they involve otherwise unexplained pain in the vulva. Vulvodynia may present as primary or secondary, appearing before or after a period of “pain-free intercourse” or provoked or unprovoked, meaning pain is elicited upon touch or presents randomly (Haefner et al., 2005; Stockdale and Lawson, 2014). Vulvodynia may also be localized to the vulvar vestibule or generalized (Haefner et al., 2005; Stockdale and Lawson, 2014). These medical terminologies, based on clinical presentation alone, may not translate to clear biological divisions of disease. Therapies that are effective for localized disease, may not work for generalized disease, and so forth. Therefore, it is imperative that studies are carefully designed with this information in mind.

Initially, vulvodynia was named vestibulitis, implying the involvement of inflammation. Tissue from affected areas clearly showed the presence of infiltrating immune cells, consistent with an inflammatory response (Prayson et al., 1995; Chaim et al., 1996; Chadha et al., 1998). However, tissue from patients without disease also showed the presence of immune cells (Halperin et al., 2005). Therefore, this disease was reclassified as vulvodynia, focusing on the characteristic of pain (Haefner et al., 2005; Bornstein et al., 2016). However, it has since been demonstrated that although the hallmarks of inflammation are present in both patients with and without vulvodynia, the relative numbers and organizations of these cells differ between cases and controls (Leclair et al., 2011; Leclair et al., 2013; Leclair et al., 2014; Falsetta et al., 2015; Foster et al., 2015; Tommola et al., 2015; Falsetta et al., 2016; Tommola et al., 2016; Falsetta et al., 2017; Falsetta et al., 2018; Zanotta et al., 2018; Falsetta et al., 2021; Falsetta et al., 2023). The painful site in patients with localized disease shows a unique inflammatory profile compared to non-painful areas of the vulva from the same patient and matched sites from controls that do not have vulvar disease (Falsetta et al., 2015; Foster et al., 2015; Falsetta et al., 2016; Falsetta et al., 2017; Falsetta et al., 2018; Falsetta et al., 2021; Bekauri et al., 2023; Falsetta et al., 2023). In essence, the vestibule of patients with vulvodynia is exquisitely sensitive to inflammatory stimuli (e.g. microbes, pathogen-associated molecular patterns), setting off a series of events that culminates in chronic pain.

Inflammation and pain are often associated. However, it was unclear how these were associated in vulvodynia until recently (Falsetta et al., 2017; Bekauri et al., 2023; Falsetta et al., 2023). Inflammation induces changes in the vulvar lipidome, specifically at painful sites, that favor inflammation and help to generate or preserve lipid signals that initiate pain signaling through transient receptor potential cation channels (TRPs), specifically subfamily V (TRPVs) (Falsetta et al., 2021; Bekauri et al., 2023; Falsetta et al., 2023). At the same time, the painful vulvar vestibule exhibits a deficiency in the production of lipids that help to abate inflammation, known as special pro-resolving mediators (SPMs). SPMs, unlike non-steroidal anti-inflammatory drugs (NSAIDs), do not impede inflammation (Serhan et al., 2015; Serhan, 2017). They are naturally produced from dietary sources of polyunsaturated fatty acids (PUFAs), and their role is to help to resolve inflammation faster and more efficiently to prevent the unintended side effects of unchecked inflammation. In localized provoked vulvodynia, there appears to be a “perfect storm” where inflammatory stimuli, potentially even the resident microflora, trigger an exaggerated immune response that results in activation of pain signaling, while sustaining inflammation to perpetuate a “never-ending” cycle of pain and inflammation (Falsetta et al., 2021; Bekauri et al., 2023; Falsetta et al., 2023) (**Figure 1**). Although more work is needed to fully understand the “key players” at a cellular level, there is now evidence of a neuro-inflammatory mechanism of disease that represents a reasonable target for the development of new therapeutics.

**Figure 1:**
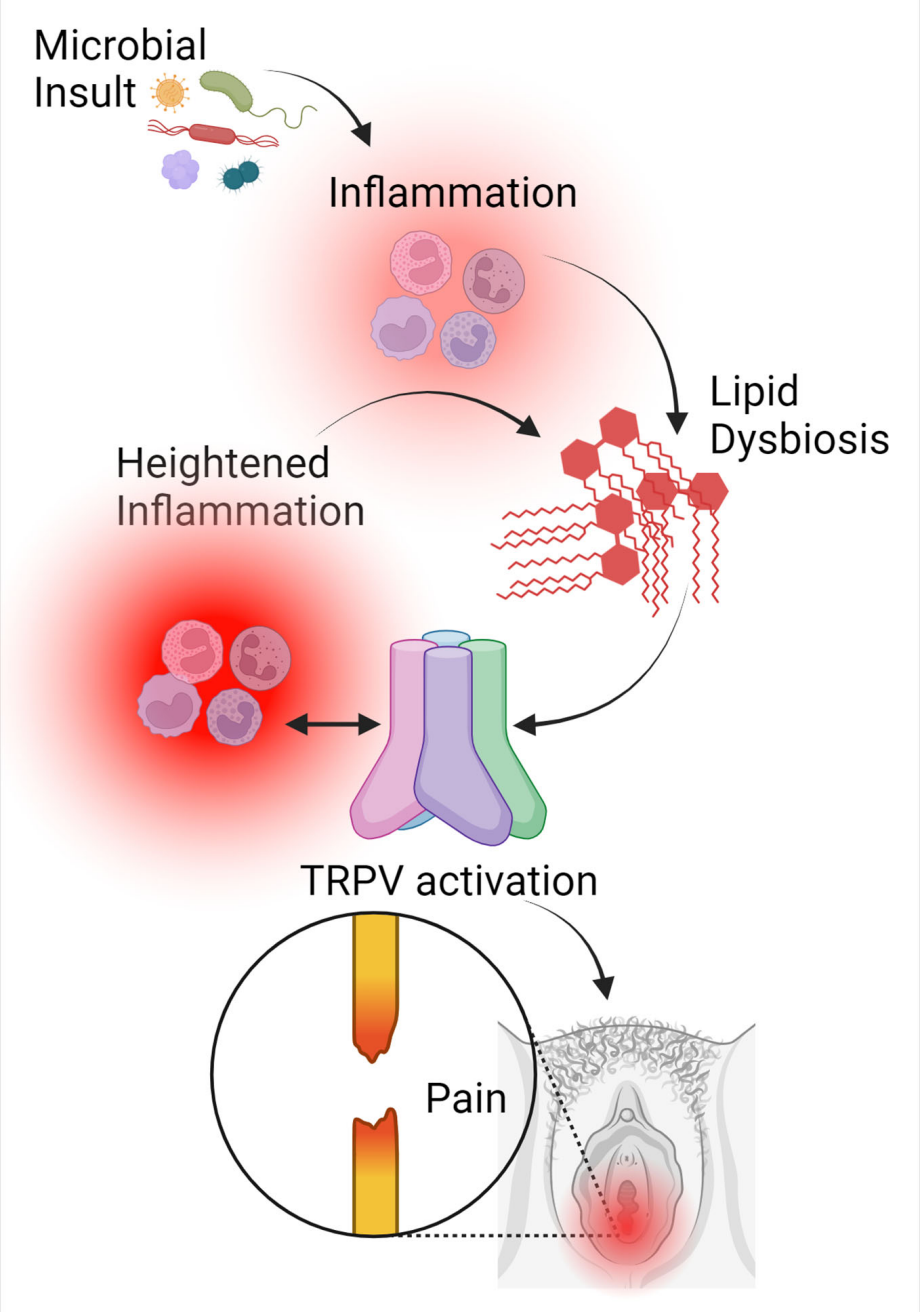
Inflammation is a catalyst for pain signaling in vulvodynia. In patients with localized provoked vulvodynia, an actual or perceived microbial insult triggers an inflammatory response at the site of pain that causes lipid dysbiosis, which favors inflammation and reduces the ability to resolve that inflammation. At the same time, levels of lipids that activate pain signaling through TRPV channels are sustained. When TRPV is activated, inflammation is further heightened, continuing to trigger lipid dysbiosis. Inflammation is both necessary and sufficient for TRPV signaling, while TRPV activation further increases inflammation. This creates a feed-forward loop where inflammation and lipid dysbiosis fuel one another, leading to chronic pain and inflammation. This figure was created using BioRender.com.

Mouse models have been optimized to test a variety of a promising new therapeutics and to further dissect these disease mechanisms (Farmer et al., 2011; Martinov et al., 2013; Arriaga-Gomez et al., 2019; Boo et al., 2019; Falsetta et al., 2021; Rangappa et al., 2021; Awad-Igbaria et al., 2022). While inflammation clearly serves a key role in at least the localized provoked disease subtype (Falsetta et al., 2015; Foster et al., 2015; Falsetta et al., 2016; Falsetta et al., 2017; Falsetta et al., 2018; Falsetta et al., 2021; Bekauri et al., 2023; Falsetta et al., 2023), there are many other factors at play that may cause, exacerbate, or sustain disease. Some of the most promising therapeutic options under investigation thus far involve modulation of this immune response, potentially through the addition of exogenous SPMs or PUFAs (Falsetta et al., 2021; Bekauri et al., 2023; Falsetta et al., 2023). However, further investigation of the underlying disease mechanisms, particularly the biological distinctions between disease subtypes, is critical for development and optimization of new therapeutic strategies. Even if an ideal therapy is on the horizon in the coming decade, it will only be useful to patients that receive a diagnosis. Prompter diagnosis is likely to reduce the incidence and impact of comorbidities, such as depression. Characterizing and harnessing the inflammatory and lipid signatures of disease could be translated to improved and objective diagnostic measures that will help alleviate the feelings of isolation and powerlessness that patients often experience. The overarching goal is to treat the disease and not simply the symptoms of that disease, while minimizing the length and extent of suffering each patient must first face.

## Author contributions

MF: Writing – original draft.
